# From eDNA to decisions using a multi-method approach to restoration planning in streams

**DOI:** 10.1038/s41598-024-64612-5

**Published:** 2024-06-21

**Authors:** A. J. Adams, C. Kamoroff, N. R. Daniele, R. L. Grasso, B. J. Halstead, P. M. Kleeman, C. Mengelt, K. Powelson, T. Seaborn, C. S. Goldberg

**Affiliations:** 1 School of the Environment, Washington State University, Pullman, WA 99164 USA; 2 Earth Research Institute, University of California, Santa Barbara, CA 93106 USA; 3 Resource Management and Science, Yosemite National Park, El Portal, CA 95318 USA; 4 Stillwater Sciences, Davis, CA 95618 USA; 5 Western Ecological Research Center, Dixon Field Station, U.S. Geological Survey, Dixon, CA 95620 USA; 6 Western Ecological Research Center, Point Reyes Field Station, U.S. Geological Survey, Point Reyes Station, CA 94956 USA; 7 Ecosystems Mission Area, U.S. Geological Survey, Modoc Hall, Sacramento, CA 95819 USA; 8 Tahoe National Forest, U.S. Forest Service, Nevada City, CA 94949 USA; 9 School of Biological Sciences, Washington State University, Pullman, WA 99164 USA; 10 School of Natural Resource Sciences, North Dakota State University, Fargo, ND 58047 USA

**Keywords:** Restoration ecology, Conservation biology

## Abstract

Reintroduction efforts are increasingly used to mitigate biodiversity losses, but are frequently challenged by inadequate planning and uncertainty. High quality information about population status and threats can be used to prioritize reintroduction and restoration efforts and can transform ad hoc approaches into opportunities for improving conservation outcomes at a landscape scale. We conducted comprehensive environmental DNA (eDNA) and visual encounter surveys to determine the distribution of native and non-native aquatic species in two high-priority watersheds to address key uncertainties—such as the distribution of threats and the status of existing populations—inherent in restoration planning. We then used these occurrence data to develop a menu of potential conservation actions and a decision framework to benefit an endangered vertebrate (foothill yellow-legged frog, *Rana boylii*) in dynamic stream systems. Our framework combines the strengths of multiple methods, allowing managers and conservation scientists to incorporate conservation science and site-specific knowledge into the planning process to increase the likelihood of achieving conservation goals.

## Introduction

Globally, translocations and reintroductions are increasingly used to counteract biodiversity losses^1–6^. Conservation translocations are often helpful for recovery of species following extirpations or genetic bottlenecks; however, attempts to implement conservation translocations have resulted in mixed success—ranging from recovery to reduced survival and repatriation failure^7–11^. The current patchwork of trial-and-error is inefficient, and a structured approach to refining reintroduction methods and planning would improve translocation outcomes^8,12–15^.

Approaching restoration and reintroduction with scientific methods and data—such as efficient survey techniques with sample sizes large enough to determine meaningful baselines—ensures effectual use of limited conservation resources and improves decision making^16^. Management decisions are frequently shrouded in uncertainty; for example, it is not always apparent why species have been extirpated from an area^17^. To reduce or explicitly incorporate uncertainties into decision-making, a well-structured, data-driven approach to planning reintroductions (and other conservation actions) is needed^16,18–20^.

Collaboration between managers and conservation scientists to incorporate conservation science and site-specific knowledge into the planning process increases the likelihood of achieving conservation goals^21^. For example, prioritizing and choosing locations for reintroductions and associated restoration efforts—such as invasive species removal—is informed by reintroduction biology^22^ as well as managers’ understanding of site-specific conditions^23,24^ and the feasibility of various management alternatives^23,25,26^. Prioritizing management alternatives based on site conditions facilitates interventions while minimizing waste and “analysis paralysis” (i.e., when problems go unaddressed while more data are collected and analyzed)^27^. Tools that explicitly facilitate managers’ decision-making process by directly addressing uncertainties and comparing alternative actions increases the likelihood of reintroduction success^19,23,26^.

Prioritizing management actions under uncertainty is paramount in the southern Sierra Nevada, USA. The Sierra Nevada encompass a biodiverse and dynamic landscape, with numerous endemic species subjected to frequent disturbances, such as flooding, landslides, drought, and wildfire, many of which are now exacerbated by climate change^28^. Thus, it is important to understand which recovery actions in this region would help meet conservation goals.

Many amphibians in the region are declining, including the stream-breeding foothill yellow-legged frog (*Rana boylii*), which is listed as endangered in the southern Sierra Nevada^29,30^. Conservation planning for *R. boylii* is complex because disease and non-native aquatic predators are among the leading causes of decline^31–34^. Non-native aquatic predators and competitors of *R. boylii* in the southern Sierra Nevada include American bullfrogs (*Lithobates catesbeianus*; hereafter “bullfrogs”) and multiple species of crayfish (*Pacifastacus leniusculus*, *Procambarus clarkii*, *Orconectes rusticus*, and *Faxonius virilis*; hereafter “crayfish”). Bullfrogs and crayfish may act as reservoir hosts for the amphibian pathogen *Batrachochytrium dendrobatidis* (Bd)^35–37^. Bullfrog presence has also been associated with increased Bd infection burdens, and in some cases, die-offs, in *R. boylii*^35^. Chytridiomycosis—the disease caused by Bd—may have contributed substantially to *R. boylii*’s historical decline, and chytridiomycosis-induced mortality events have been observed contemporarily in the species^17,35,38^. Non-native predator eradication programs—such as those removing non-native fish and bullfrogs—have been successful in restoring some Sierra Nevada aquatic habitats^39,40^. These eradication initiatives have laid the groundwork for a surge in ranid reintroduction efforts^4,7,24,41–44^, presenting an important opportunity to closely examine restoration methods and prioritization.

Many tools are available to help plan management actions. Although geographical information system tools and remote sensing can assist in habitat evaluation at a coarse scale, developing information about resource quality at the local scale requires time in the field^45^—whether through field surveys, direct observation, and/or sample collection—which may include environmental DNA (eDNA). Environmental DNA is trace DNA found in the water column and other substrates that can be used to detect species^46,47^. The relatively low cost, minimal invasiveness, simple field collection, and rapid processing of samples make eDNA detection methods well-suited for monitoring aquatic wildlife species^39,48,49^. The high sensitivity of eDNA analysis also allows for detection of invasive, cryptic, rare, or even presumed-extinct species^50–53^ that may escape other methods of detection, such as visual encounter surveys (VES). Environmental DNA methods can be used to test for the presence of *R. boylii*, and therefore help designate sites as either occupied and a potential source population, or as having restoration and recipient potential^54^. The sensitivity and geographic specificity of eDNA also make it an effective tool for focusing targeted efforts on invasive aquatic species eradications and monitoring for their reestablishment^39,55^.

*Rana boylii* are highly detectable in streams using eDNA methods^51^, as are the invasive bullfrogs and crayfish^56,57^ affecting *R. boylii* in the southern Sierra Nevada^35,58,59^. Invasive species removal efforts and conservation translocations of *R. boylii* to areas from which they have been extirpated is urgent in the region. In particular, the Tuolumne and Merced River watersheds were chosen as high priority conservation areas by management agencies due to the opportunities for restoration with an abundance of publicly accessible land and climate refugia^24,60^. Genetic variability of remaining *R. boylii* populations in the region is high^61^, providing good potential sources for translocations and reintroductions. Much uncertainty remains, however, in the distribution of *R. boylii* and invasive species in the southern Sierra Nevada. Therefore, combining site-specific species occurrence data with an explicit method for prioritizing management actions at sites would improve recovery of *R. boylii* in the region.

In this study, we applied a mixed methods approach to addressing uncertainties in the habitat restoration and reintroduction of *R. boylii* in the southern Sierra Nevada. Specifically, we addressed occurrence uncertainties for *R. boylii*, bullfrogs, crayfish, and Bd at the landscape scale by combining eDNA sampling with VES. Results of these surveys were then used to determine appropriate recovery actions through the development of a decision framework in concert with a prioritized menu of site-specific restoration actions. Our approach combines multiple species with multiple methods (i.e., eDNA, VES, and decision framework development) to directly inform decision making. The questions we addressed were (1) What is the distribution of *R. boylii* in the study area; (2) What is the distribution of biological threats to *R. boylii* (i.e., non-native aquatic predators and a pathogen) in the study area; and (3) How can this information be used to discern and prioritize restoration actions for *R. boylii* in a dynamic stream system?

## Methods

### Study area

**Figure 1:**
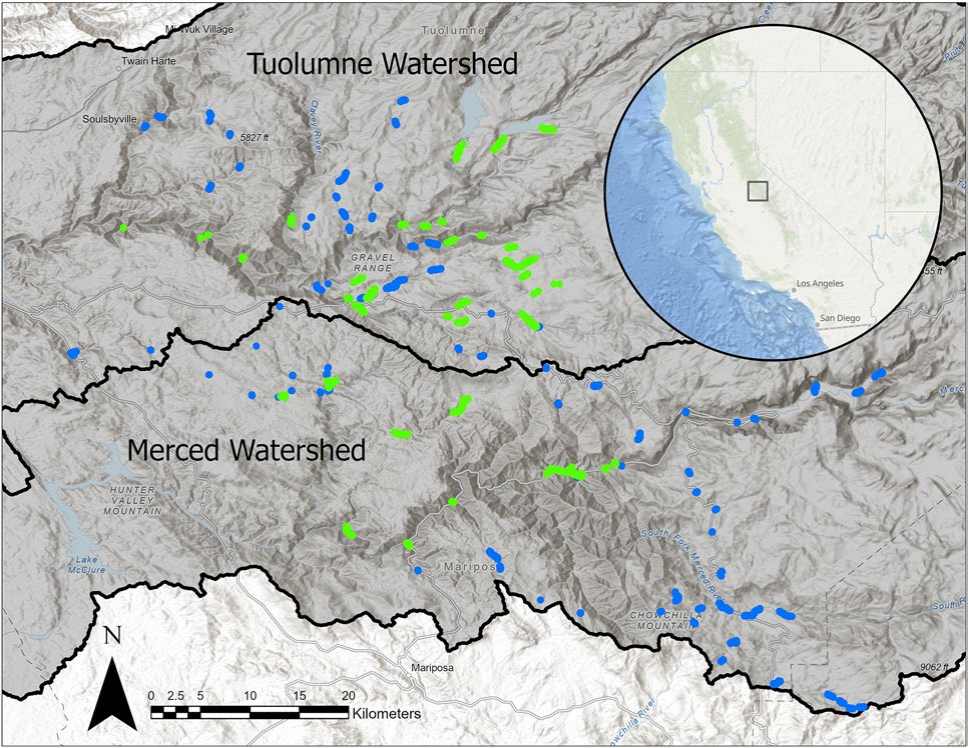
Map of survey transects in the southern Sierra Nevada, California, USA, conducted June – September 2020 (green) and 2021 (blue).

The southern Sierra Nevada presents unique challenges and opportunities for prioritizing *R. boylii* recovery. Encompassing approximately 9540 km^2^, the Tuolumne and Merced River watersheds (Fig. 1) fall under primarily federal jurisdiction, facilitating the ongoing implementation of recovery actions and long-term monitoring as well as presenting opportunities to leverage professional expertise and funding. Conventional VES for *R. boylii* are challenging in this region’s rugged landscapes, where the terrain prohibits or reduces access to, and visibility in, many streams.

### Site selection

To facilitate broad-scale sampling across the Tuolumne and Merced River watersheds, we selected focal sites (i.e., stream locations for which individual management action decisions can be made) to survey based on historical and contemporary *R. boylii* occurrence data (e.g., the California Natural Diversity Database^62^). We obtained historical and contemporary *R. boylii* and non-native species occurrence information from local, state, and federal wildlife and land management agencies (including the Bureau of Land Management, U.S. Forest Service, U.S. National Park Service, California Department of Fish and Wildlife, and U.S. Geological Survey). Using the *R. boylii* localities, we identified the sites that had the highest restoration potential, based on (1) accessibility, (2) habitat suitability, and (3) most recent observations. All sites needed to be accessible for surveys, future monitoring, and restoration activities. Therefore, we only considered sites if they were accessible via road or trail or if the landscape was amenable to cross-country travel. We also only considered restoration sites that were publicly owned, as private land ownership could hinder future monitoring efforts or restoration activities. We chose sites that were considered suitable based on a *R. boylii*-specific habitat model of connections between blocks of protected lands in the Sierra Nevada^63^. Of the sites that were considered suitable and accessible, we prioritized sites with recent observations, wherein sites where frogs were presumed extant (post-2006) were prioritized over historical (prior to 2006) sites. We identified 57 sites within 9 HUC-8 level sub-watersheds: North Fork Merced River (N = 5); South Fork Merced River (N = 12); main stem Merced River (N = 16); South Fork Tuolumne River (N = 5); Middle Tuolumne River (N = 4); North Fork Tuolumne River (N = 2); and main stem Tuolumne River (N = 13).

### Sample collection and analysis

We conducted VES and eDNA surveys June–September 2020 and 2021 to target when flows are low and eDNA from the focal species is more likely to be concentrated and therefore more detectable^51,64^. Because we designed the study to be comprehensive (i.e., for maximum detection of the target species in specific management areas of interest), we surveyed throughout all sites of interest rather than conducting random sampling. Water samples were collected using 5-µm polyethersulfone self-desiccating filters and a backpack-based pump system (Smith-Root, Vancouver, Washington, USA)^65,66^. The 5-µm pore size was selected to allow for large volumes of water to be filtered continuously. Because eDNA has limited transport in small streams^51^, we surveyed by sampling continuously while walking slowly upstream in the water, with the filter extended out on a telescoping pole (≥ 2 m), collecting water from pools and the main channel. Filter packs were sealed back in their packaging and stored in the dark at ambient temperature. Pump sample volume was set to 0.5 L/min, using a 190-mL offset in the sample volume quantification to account for the water held in the input tube and pump, per the manufacturer’s instructions^67^. Filter samples were collected as a single sample per transect, and transects were made continuous by replacing each filter when it clogged or the pump broke prime (i.e., lost effective vacuum pressure), whichever came first. Multiple filters were frequently collected within each stream (mean = 4.3; range = 1–22 filters). Fresh nitrile gloves were worn when handling each filter housing. Field blanks were collected by filtering 1 L of distilled water at the end of each field day prior to decontamination of equipment. All external gear (i.e., waders, boots) was decontaminated with a 10% bleach solution when moving between streams to avoid spreading microorganisms (such as Bd) between sites. The backpack sampler was also decontaminated internally with a 2% bleach solution, per the manufacturer’s instructions^67^. Two independent-observer visual encounter surveys were also conducted within each eDNA stream transect, so that three total replicate surveys (one eDNA and two VES) were conducted within each stream reach. The VESs were conducted while wading in-stream wherever possible, because this is the best vantage point from which to see *R. boylii* basking on rocks or the edges of rocks in the stream. The rugged terrain in many areas also makes the banks impassable by foot. The VESs occurred at least 30 min after eDNA sample collection^68–70^, either the same day or within 48 h of eDNA sampling. Stream water temperature was measured using a hand-held thermometer at the beginning of each survey. At two sites in 2021, we collected Bd swabs from the skin of early post-metamorphic *R. boylii* following standardized procedures^71^.

Because the primary objective of this study was to determine which actions would be most beneficial in specific areas, we collected and analyzed the data in a way that could inform these decisions. Previous studies have demonstrated very high probabilities for eDNA detection of our target species^39,51,72,73^. We paired eDNA with VES for the additional ability to detect species that could be missed with eDNA methods. Given the purpose of the study, we did not choose sites to sample randomly or attempt to draw inference to unsampled areas. Instead, we aimed to survey primary places of interest that had the highest conservation and restoration potential (refer to Site Selection, above), in order to inform which recovery actions would be the most beneficial to attempt at specific sites.

We extracted DNA from samples using the Qiashredder/DNeasy protocol of Goldberg et al.^53^ in a restricted access laboratory dedicated to low-quantity samples, and analyzed samples using published quantitative polymerase chain reaction (qPCR) assays for *R. boylii*^51^, American bullfrog (*L. catesbeianus*^74^), and the amphibian chytrid fungus (*B. dendrobatidis*; Bd^75^). We also developed and applied an assay for the signal crayfish (*P. leniusculus*; Supplementary Molecular Methods; Supplementary Table S1). Because Bd was only detected in one filter in 2020, we did not assay for Bd in all filter samples in 2021. In 2021, we only tested the water filters for Bd in the streams where we were collecting Bd swabs at the same time. We collected the eDNA samples immediately before collecting the Bd swabs. In 2020, we included surveys and assays for northwestern pond turtle (*Actinemys marmorata*) and three trout species to determine whether *A. marmorata* and native trout may be positive ecological indicators for *R. boylii*, and the extent to which non-native trout may be *R. boylii* predators. However, due to funding constraints, we did not test for *A. marmorata* or trout species in the 2021 samples and they were therefore not included in this analysis (refer to Supplementary Information).

Environmental DNA and Bd swab samples were initially analyzed in triplicate and considered positive if they tested consistently positive across the three wells. Samples that produced inconsistent results (1 or 2 positive) were rerun in triplicate and considered positive if they tested positive in ≥ 1 wells in both triplicate runs. An internal positive control (ThermoFisher Scientific, Waltham, Massachusetts, USA) was included with each assay, and samples were cleaned with a OneStep™PCR Inhibitor Removal Kit (Zymo Inc., Irvine, California, USA) if the Cq for the internal control was delayed > 3 cycles. If samples continued to test as inhibited after treatment, they were further diluted 1:10 and then 1:100. Any sample needing 1:100 dilution that tested negative was considered inconclusive and excluded from the study. Quantitative standards were run in duplicate for each species, consisting of either DNA samples derived from tissue from external skin, diluted 1:1000 through 1:1,000,000 in QuantiTect Nucleic Acid Dilution Buffer (Qiagen®), or gblock™ standards (Integrated DNA Technologies, Coralville, Iowa, USA), in a tenfold series dilution 10,000 to 10 copies in QuantiTect Nucleic Acid Dilution Buffer (Qiagen; refer to Supplementary Molecular Methods). All eDNA and VES data were managed, analyzed, or visualized using Microsoft Excel, ArcGIS Pro, and R^76–78^.

### Decision support tool development

At the request of the U.S. Fish and Wildlife Service, we conducted an assessment of feasible *R. boylii* management actions based on the VES and eDNA survey results. After evaluating sites of interest for the threat of non-native predators with eDNA and VES, we identified and prioritized feasible *R. boylii* recovery actions for each site. This assessment was done in consultation with management agency stakeholders, including U.S. National Park Service staff that have conducted California red-legged frog (*Rana draytonii*) translocations to Yosemite Valley^4,24,44^, as well as U.S. Forest Service staff that have conducted monitoring at *R. boylii*-occupied sites.

**Figure 2:**
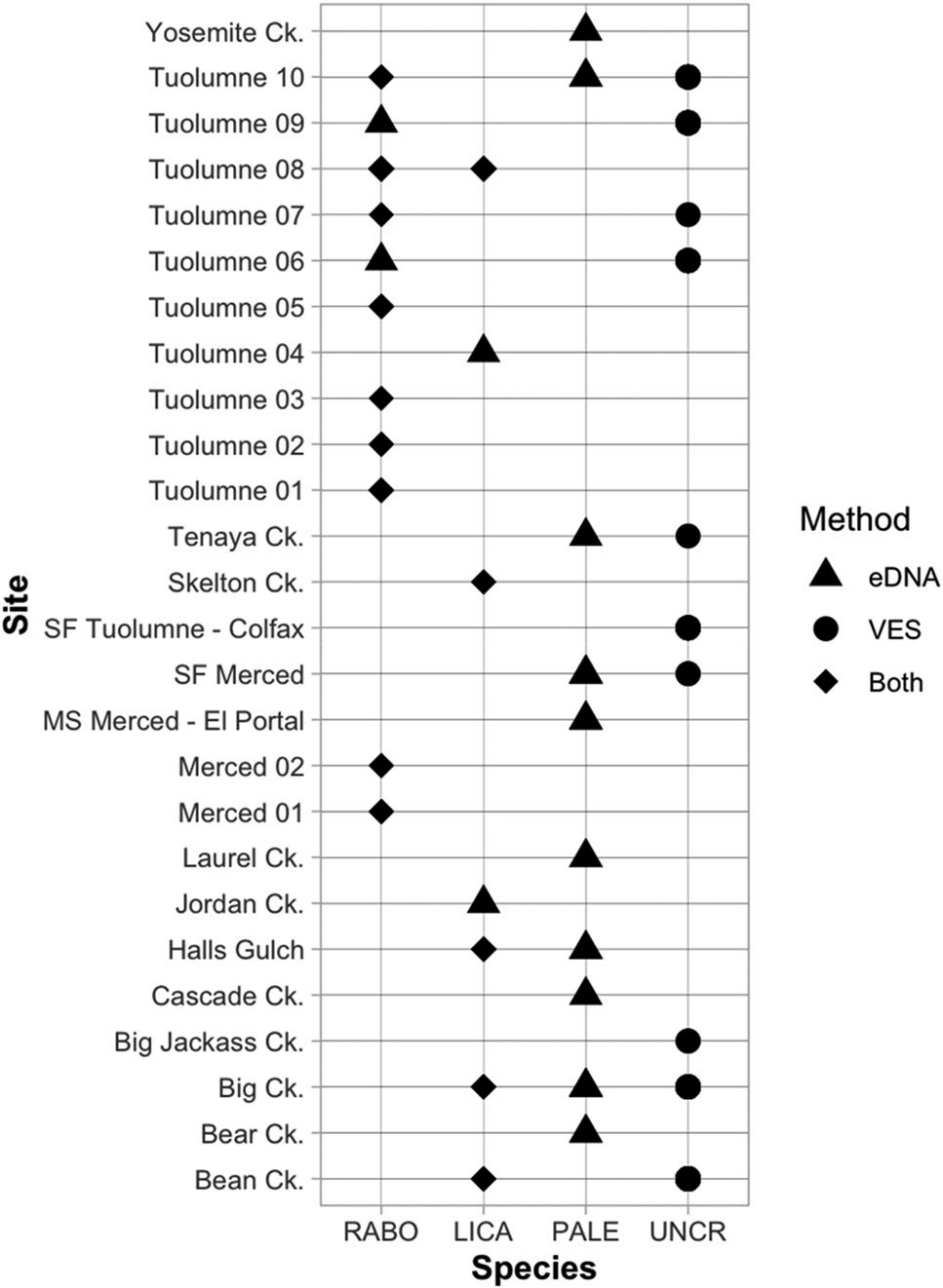
Environmental DNA (eDNA) and visual encounter survey (VES) detections for the foothill yellow-legged frog and non-native aquatic predator species that threaten its survival and recovery. LICA = American bullfrog (*Lithobates catesbeianus*); PALE = signal crayfish (*Pacifastacus leniusculus*); RABO = foothill yellow-legged frog (*Rana boylii*); UNCR = unknown crayfish species. Signal crayfish were detectable only with eDNA; “unknown crayfish” were detected only with VES. Numbered sites represent streams where *R. boylii* are present, to protect the exact locations of this endangered species. SF = South Fork; MS = main stem; Ck. = creek.

To assess the suitability of specific management options at different sites, we first placed the species detection data in a site-by-species matrix, focusing specifically on *R. boylii* and non-native predator occurrence, as these are the primary species of concern for restoration decision making (Fig. 2). Each site was then assigned a status category based on the species detected at that site: *R. boylii* present without non-native predators (category “A”); *R. boylii* present with non-native predators (“B”); *R. boylii* absent without non-native predators (“C”); and *R. boylii* absent with non-native predators (“D”) (Supplementary Box 1).

**Figure 3:**
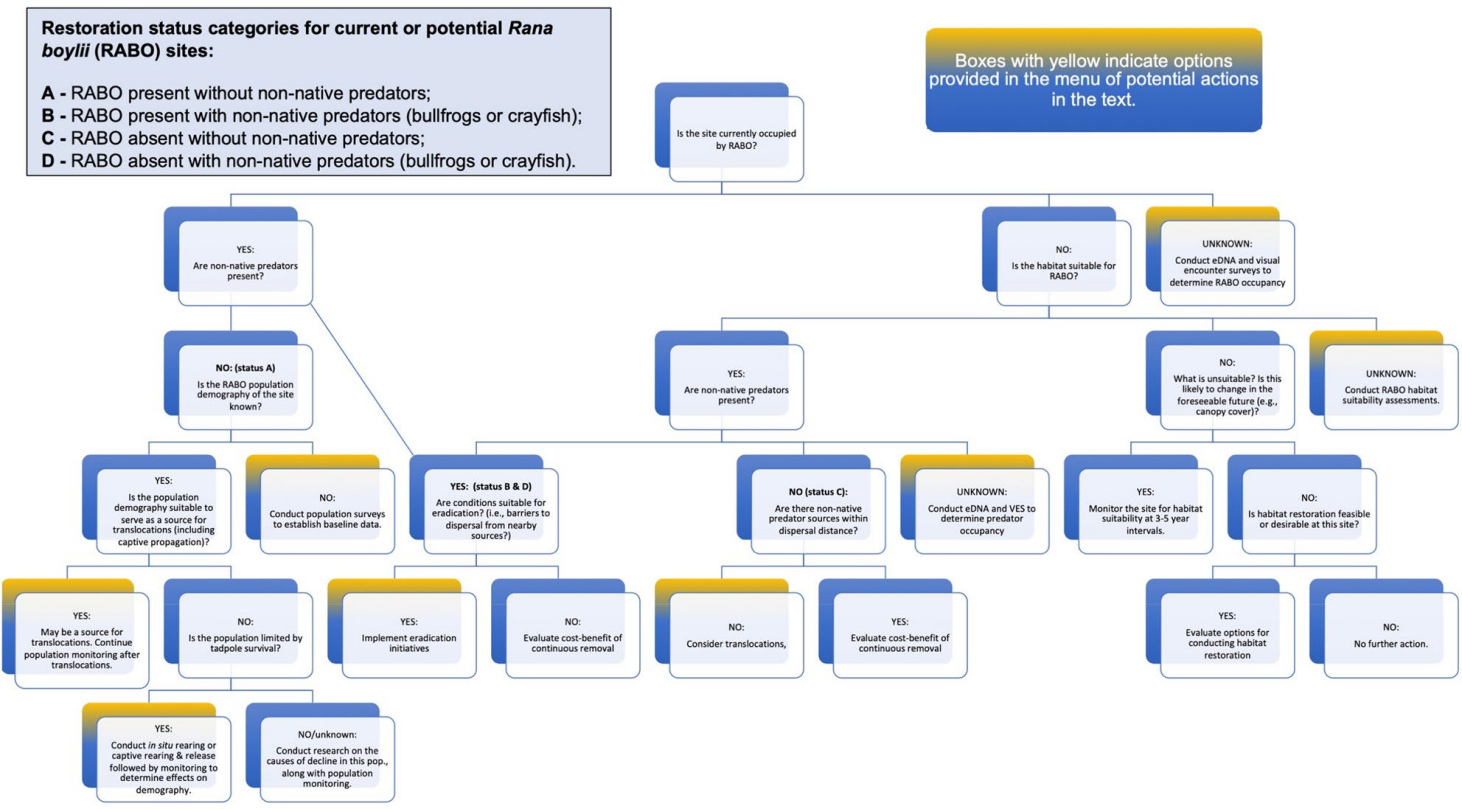
Decision tree for potential *Rana boylii* conservation actions.

At the site level, status categories were then applied to a menu of research needs and plausible actions under consideration by management agencies (“potential management actions”). Potential management actions included: habitat assessments; demographics and baseline monitoring; bullfrog and crayfish control; species detections/surveys; in situ rearing/captive rearing and release; and reintroductions or translocations (Supplementary Information). The menu, informed by the restoration categories, was placed within the context of a decision tree (Fig. 3) to provide a stepwise framework to evaluate site-specific management actions for *R. boylii* conservation, depending on priority and threat. For example, at a site where *R. boylii* is present without non-native predators (category A), the management actions could include collecting demographic baseline monitoring data to determine the status of the *R. boylii* population, and determining whether captive propagation (whether in situ [i.e., in-stream] captive propagation and reintroduction) or translocations from another site or ex situ captivity are appropriate (Supplementary Table S2).

Finally, sites were assigned to priority classifications based on both status category and site-specific constraints (Table 1; Supplementary Table S2). The highest-priority sites are those where potential near-term management actions are expected. This may include sites where bullfrogs and crayfish currently threaten existing *R. boylii* populations, or where these species are within dispersal distance of *R. boylii* populations and therefore may pose an imminent invasion threat. High priority sites are also those where *R. boylii* occurs and demographic data would be needed to determine whether the population may serve as a source population, or whether captive propagation would be needed to bolster existing populations. One high-priority site (Merced 03; refer to Supplementary Table S2) has suitable habitat for *R. boylii* and is therefore a suitable translocation recipient site. Moderate-priority sites are those where no bullfrogs or crayfish were detected, but further evaluation would be needed for *R. boylii* reintroduction feasibility. Low priority sites are those where bullfrogs, crayfish, or both were detected and *R. boylii* are absent, and the site is not currently within dispersal distance of suitable translocation sites. In addition, a site may be regarded as low priority even if bullfrogs and crayfish were not detected if other factors make the site unsuitable for any of the potential management actions. For example, Fern Spring is currently unsuitable for reintroductions because it is in an extremely high-traffic area; Tuolumne 03, although occupied by *R. boylii*, is a low priority for any of the potential actions because of its remote location, which both protects it from the threat of non-native predators being introduced but also makes most of the potential management actions infeasible (refer to Supplementary Table S2).

**Table 1:** Sample management actions and prioritization of sites by status for the North Fork Merced River.

Site	Status	Management action(s)	Comments	Priority
Bean Creek	D	BC	Bullfrogs & crayfish detected. No barriers to dispersal to nearby *R. boylii* sites Merced 01 and Merced 02. High priority to control bullfrogs and crayfish nearest the *R. boylii*-occupied sites	High
Deer Lick Creek	C	HA	No bullfrogs or crayfish detected. Evaluate whether suitable for reintroduction with habitat assessments	Moderate
Jordan Creek	D	BC	Bullfrogs detected, and no barriers to dispersal to two nearby *R. boylii*-occupied sites. High priority to control bullfrogs and crayfish nearest these sites	High
Merced 01, Merced 02	A	DG; SD	Demographic data needed on *R. boylii* population to determine if captive propagation is warranted. Population likely threatened by bullfrogs and crayfish in two nearby creeks. Continue sampling (species detections) for bullfrogs and crayfish in order to monitor for invasions into this site	High

A complete table of all sites is provided in the Supplementary Information. Status categories: A = *Rana boylii* present without non-native predators; B = *R. boylii* present with non-native predators (bullfrogs or crayfish); C = *R. boylii* absent without non-native predators; D = *R. boylii* absent with non-native predators (bullfrogs or crayfish). Potential management actions: HA = habitat assessments; DG = demographics/baseline monitoring; BC = bullfrog or crayfish removal; CP = captive propagation, which could include in situ rearing in the habitat or captive rearing at a qualified facility followed by release to the same site; SD = species detection monitoring (i.e., continued environmental DNA [eDNA] and visual encounter survey [VES]); TR = translocations.

## Results and discussion

### Species detections

In total, we surveyed 57 sites and 186 km of stream (Fig. 1), filtering 2690 L of water through 367 eDNA filters (mean 47.2 L per site; mean 6.4 filters per site) in addition to 76 field blanks. All field blanks were assayed for all species associated with the year of sampling and tested negative. *Rana boylii* were detected at 11 sites and non-native predators (bullfrogs, crayfish, or both) co-occurred with *R. boylii* at five of these sites (Fig. 2). Neither *R. boylii* nor non-native aquatic predators were detected at 31 out of the 57 sites (54%). Mean stream temperature during eDNA sample collection was 18.16 °C ± 4.83 standard deviation (SD).

We detected Bd in a single filter (two out of three replicates, > 33.7 internal transcribed spacer [ITS] copies/L) in 2020 (site Tuolumne 02). We detected *R. boylii* in this stream as well, but not from the same filter. It is unlikely that the non-detection of Bd eDNA at other sites represents the absence of Bd at these sites, as Bd has been detected in *R. boylii* swabs from other sites in the study area (AJA, unpublished, 2020–2021). In 2021, we detected Bd in six swabs collected from 11 *R. boylii* metamorphs in two different streams (one Bd positive in Tuolumne 08 and five in Merced 01). However, no Bd eDNA was detected from either of these streams. Swab-detected Bd prevalence and mean infection burden were high in *R. boylii* in Merced 01 (100% and 1.54 × 10^6^ copies, respectively). The corresponding eDNA extract from the filter collected in the same stream immediately prior to swabbing was still inhibited after treatment for inhibitors and a 1:10 dilution of those treated extracts. Therefore, only a very strong signal would have been able to be detected from this sample. The high level of inhibition is likely because of the low streamflow when this sample was collected, increasing the likelihood of PCR-inhibitory compounds getting into the filter. It is not clear why we did not detect Bd in the broader sampling of these stream systems, but it seems unlikely that these results reflect true Bd prevalence. In a nearby lentic system, Bd eDNA detection was sensitive enough to predict chytridiomycosis outbreaks^79^, and Bd was detected in *R. boylii* eDNA samples from streams in the central Sierra Nevada^54^. Stream eDNA does not spread far from a source^54^ and detection requires continuous input^80^. If Bd shedding is temporally patchy (e.g., if many individuals in a population respond to temperature conditions with certain basking behaviors^81,82^), detection in lotic systems may be rare when infected animals are at low densities. Bd did not play a major role in the decision support tool criteria because the data for it were so sparse; however, the high Bd loads on swabs collected at site Merced 01 have been communicated to the relevant management agencies.

*Actinemys marmorata* (the native turtle that we sampled for in 2020) and *R. boylii* frequently co-occur throughout their range^83^, and we detected *A. marmorata* with either eDNA or VES in four of eight *R. boylii*-occupied streams. This indicates that some of these sites are more intact systems and that the two species have overlapping ecological requirements, further helping to prioritize sites for reintroduction. Therefore, restoring habitat for *R. boylii* may also benefit the conservation of *A. marmorata*, which is proposed for listing under the U.S. Endangered Species Act^84^.

The benefits of eDNA sampling in conjunction with VES varied by species. In one instance, the survey crew identified a small frog as *R. boylii* while eDNA analysis only detected bullfrogs at that site. After the coauthors—who have decades of field experience with the species in question—examined the photographs taken by the surveyors, it became clear that the frog was indeed a juvenile bullfrog, not *R. boylii*. These two species can be easily mistaken when the individual is small, exemplifying the advantages to having a multi-method design that combines natural history knowledge and observation with assay-driven eDNA techniques. In addition, we detected *A. marmorata* at four more streams with eDNA than with VES (eDNA = 9; VES = 5), indicating that eDNA is a much more sensitive method for *A. marmorata* detection than VES. This result is consistent with recent findings from farther north in California^85^. Only 20% of visual “unknown crayfish” detections and *P. leniusculus* eDNA detections overlapped (Fig. 2), indicating that there is at least one additional crayfish species present in the study area that was not assayed for, and that *P. leniusculus* is more difficult to detect visually than the other species. The other crayfish species known to occur in the Merced and Tuolumne River watersheds include the red swamp (*P. clarkii*), rusty (*O. rusticus*), and virile (*F. virilis*) crayfish, all of which are outside of their native range in the study area^86^. These individual species are not identifiable in the field during VES surveys, often requiring gonopod examination under a microscope^87^. Finally, eDNA could likely provide information on pathogens that is not available from field observations; however, in this case we had almost no eDNA detection of Bd, which seems unlikely to reflect true Bd prevalence in these streams and differs from what we know of Bd prevalence in local lakes^79^.

### Advancing the use of eDNA to landscape-scale decision making

Through the process of ascertaining site-appropriate restoration actions based on the eDNA and VES data, we identified 15 high-priority sites, 30 moderate-priority sites, and 12 low-priority sites in the study area (Supplementary Table S2). We detected bullfrogs and crayfish successfully using both eDNA and VES, and the use of eDNA provided an early warning system for bullfrog and crayfish invasions into *R. boylii*-occupied streams. At an *R. boylii*-occupied site where bullfrogs had previously been eradicated (Tuolumne 04), bullfrogs were not detected during routine VES monitoring and eDNA sampling conducted from 2017 to 2019 (R. Grasso, Yosemite National Park, written communication, 2021). We did not detect bullfrogs with eDNA or VES at this site in 2020; however, in 2021, we detected bullfrogs there with eDNA, but not VES. Bullfrogs were detected with eDNA, but not VES, at site Tuolumne 08 for the first time in 2021—in a portion of the stream where only *R. boylii* were detected in 2020. At Jordan Creek—a site with previously known bullfrog populations—bullfrogs were detected with eDNA but not VES (Fig. 2). Similarly, *P. leniusculus* eDNA was detected at six sites where the species was not detected visually (Fig. 2). These results highlight the importance of repeated, multi-year sampling for non-native species invasions in dynamic stream landscapes, for which eDNA sampling appears to be well-suited.

Using an effective monitoring strategy is critical when managing species in dynamic systems faced with ongoing threats compounded by climatic extremes. In drought years, reduced streamflows facilitate bullfrog breeding in reaches of stream that they have not previously been able to occupy in wetter years^35^. In the Sierra Nevada, signal crayfish populations are also partly regulated by streamflow: artificial impoundments provide refugia that facilitate upstream repopulation after scouring from high-flow events^88^. Similar phenomena have been observed with bullfrogs in central coastal California, where drought facilitated the movement of Bd-carrying bullfrogs to *R. boylii*-occupied streams, likely triggering observed chytridiomycosis outbreaks and the first record of chytridiomycosis-induced mortality in *R. boylii*^35^.

We leveraged a fine-scale understanding of the dynamics between *R. boylii* and non-native aquatic predators to inform reintroduction decision making and management action prioritization. Without field observations and survey efforts, and only using existing range maps and knowledge, we would not have had the information necessary to inform the planning and prioritization of on-the-ground management actions. For example, the identification of new bullfrog invasions into *R. boylii*-occupied sites has clarified dynamics between drought and potential invasive species control actions when *R. boylii* and bullfrogs occur in proximity. In addition, identification of suitable sites for *R. boylii* demographic studies where its most pressing threats are absent has increased the likelihood that these areas can be prioritized by conservation managers.

## Conclusion

We applied a multi-method approach to a data-poor, landscape-scale conservation challenge: identifying and prioritizing potential management alternatives for an endangered species in a dynamic stream system. Our research advances the integration of eDNA technology directly into conservation decision-making. Our continuous in-stream sampling technique was used for the first time at a landscape scale, and enabled eDNA detection of sparsely distributed species. This new sampling technique is powerful, both for uncovering small remnant populations of rare, imperiled species and for early eDNA detection of invasive species so they can be mitigated early in the invasion process. Our combined approach—which incorporated eDNA-derived results with a decision framework—could culminate in better-informed reintroduction decisions that streamline efforts, reduce costs, and improve the success of reintroductions and associated management efforts in the southern Sierra Nevada.

The decision support tool additionally gives managers the ability to prioritize actions based on species occupancy and site-specific criteria that can be easily altered to meet shifting management needs as more information becomes available.

To guide additional landscape-scale management actions in the future, our occurrence results can be incorporated into species distribution models to predict community responses to climate change^89^. Because the Sierra Nevada region is increasingly recognized as an important area for climate change refugia^90–93^, some portions of the ecoregion may provide future habitat for species needing cooler and wetter conditions^60^. This study provides a pathway for informed management of a declining freshwater species in an uncertain future and demonstrates how fine-scale, multi-species data obtained with eDNA can be used to prioritize actions at a landscape scale.

### Supplementary Information

Supplementary Information.

## Data Availability

The DNA sequence data generated during the current study are available in the GenBank repository [accession numbers OQ835382-OQ835460; submission number SUB13061766; https://submit.ncbi.nlm.nih.gov/subs/?search=SUB13061766; release date 24 April 2023]. The remaining data generated and analyzed are not publicly available due to the sensitive nature of localities of an endangered species, but are available from the corresponding author on reasonable request.

## References

[1] Seddon PJ, Griffiths CJ, Soorae PS, Armstrong DP (2014). Reversing defaunation: Restoring species in a changing world. Science.

[2] Nguyen AM, Halstead BJ, Todd BD (2024). Effect of translocation on home range and movements of giant gartersnakes. Global Ecol. Conserv..

[3] Knapp RA (2023). Evolutionary rescue and reintroduction of resistant frogs allows recovery in the presence of a lethal fungal disease. bioRxiv.

[4] Adams AJ, Bushell J, Grasso RL (2022). To treat or not to treat? Experimental pathogen exposure, treatment, and release of a threatened amphibian. Ecosphere.

[5] Moorhouse TP, Gelling M, Macdonald DW (2009). Effects of habitat quality upon reintroduction success in water voles: Evidence from a replicated experiment. Biol. Conserv..

[6] McFadden, M., Hunter, D., Harlow, P., Pietsch, R. & Scheele, B. in *Global re-introduction perspectives: Additional case studies from around the globe* (ed P. S. Soorae) 77–80 (IUCN/SSC Re-introduction Specialist Group, 2010).

[7] Knapp RA (2016). Large-scale recovery of an endangered amphibian despite ongoing exposure to multiple stressors. Proc. Natl. Acad. Sci..

[8] Mendelson JR, Whitfield SM, Sredl MJ (2019). A recovery engine strategy for amphibian conservation in the context of disease. Biol. Conserv..

[9] Howell PE, Hossack BR, Muths E, Sigafus BH, Chandler RB (2016). Survival estimates for reintroduced populations of the Chiricahua leopard frog (*Lithobates chiricahuensis*). Copeia.

[10] Fellers GM, Bradford DF, Pratt D, Wood LL (2007). Demise of repatriated populations of mountain yellow-legged frogs (*Rana muscosa*) in the Sierra Nevada of California. Herpetol. Conserv. Biol..

[11] Seddon PJ (2010). From reintroduction to assisted colonization: Moving along the conservation translocation spectrum. Restor. Ecol..

[12] Brignon WR, Peterson JT, Dunham JB, Schaller HA, Schreck CB (2017). Evaluating trade-offs in bull trout reintroduction strategies using structured decision making. Can. J. Fish. Aquat. Sci..

[13] van Heezik Y, Seddon PJ (2018). Animal reintroductions in peopled landscapes: Moving towards urban-based species restorations in New Zealand. Pacific Conserv. Biol..

[14] Venesky MD, Mendelson JR, Sears BF, Stiling P, Rohr JR (2012). Selecting for tolerance against pathogens and herbivores to enhance success of reintroduction and translocation. Conserv. Biol..

[15] White TH (2015). Improving reintroduction planning and implementation through quantitative SWOT analysis. J. Nat. Conserv..

[16] Ewen JG, Soorae PS, Canessa S (2014). Reintroduction objectives, decisions and outcomes: global perspectives from the herpetofauna. Anim. Conserv..

[17] Adams AJ, Pessier AP, Briggs CJ (2017). Rapid extirpation of a North American frog coincides with an increase in fungal pathogen prevalence: Historical analysis and implications for reintroduction. Ecol. Evol..

[18] Canessa S (2020). Risk aversion and uncertainty create a conundrum for planning recovery of a critically endangered species. Conserv. Sci. Pract..

[19] Converse SJ, Moore CT, Folk MJ, Runge MC (2013). A matter of tradeoffs: Reintroduction as a multiple objective decision. J. Wildl. Manag..

[20] McCarthy, M. A., Armstrong, D. P. & Runge, M. C. in *Reintroduction Biology* (ed D.P. Armstrong J.G. Ewen, K.A. Parker, P.J. Seddon) 256–289 (2012).

[21] Toomey AH, Knight AT, Barlow J (2017). Navigating the space between research and implementation in conservation. Conserv. Lett..

[22] IUCN. Guidelines for reintroductions and other conservation translocations. 72 (IUCN/SSC Reintroduction Specialist Group, Gland, Switzerland and Cambridge, United Kingdom, 2013).

[23] Pritchard RA (2022). Identifying cost-effective recovery actions for a critically endangered species. Conserv. Sci. Pract..

[24] Adams AJ, Grasso RL, Mazur RL (2023). Safe harbor: translocating California red-legged frogs to a climate refuge in Yosemite National Park. Anim. Conserv..

[25] Armstrong DP, Seddon PJ (2008). Directions in reintroduction biology. Trends Ecol. Evol..

[26] Taylor G (2017). Is reintroduction biology an effective applied science?. Trends Ecol. Evol..

[27] Fuller AK, Decker DJ, Schiavone MV, Forstchen AB (2020). Ratcheting up rigor in wildlife management decision making. Wildl. Soc. Bull..

[28] Millar, C. I. Sierra Nevada Ecosystem Project. Sierra Nevada Ecosystem Project, Final Report to Congress, Vol. I, Assessment Summaries and Management Strategies, Centers for water and Wildland Resources, Report No. 36, University of California, Davis, California. Cooperative report of the PSW Research Station, PSW Region, USDA, for the Sierra Nevada Framework Project, Sacramento, CA. (1996).

[29] California Fish and Game Commission. Notice of findings for foothill yellow-legged frog (*Rana boylii*). 13 (California Department of Fish and Wildlife, Sacramento, CA, 2020).

[30] U.S. Fish and Wildlife Service. Endangered and Threatened Wildlife and Plants; Foothill Yellow-Legged Frog; Threatened Status With Section 4(d) Rule for Two Distinct Population Segments and Endangered Status for Two Distinct Population Segments. 59698–59727 (2023).

[31] Drost CA, Fellers GM (1996). Collapse of a regional frog fauna in the Yosemite area of the California Sierra Nevada, USA. Conserv. Biol..

[32] Knapp RA, Matthews KR (2000). Non-native fish introductions and the decline of the mountain yellow-legged frog from within protected areas. Conserv. Biol..

[33] Moyle PB (1973). Effects of introduced bullfrogs, *Rana catesbeiana*, on the native frogs of the San Joaquin Valley California. Copeia.

[34] Briggs CJ, Vredenburg VT, Knapp RA, Rachowicz LJ (2005). Investigating the population-level effects of chytridiomycosis: An emerging infectious disease of amphibians. Ecology.

[35] Adams AJ (2017). Extreme drought, host density, sex, and bullfrogs influence fungal pathogen infection in a declining lotic amphibian. Ecosphere.

[36] Schloegel LM (2010). The North American bullfrog as a reservoir for the spread of *Batrachochytrium dendrobatidi*s in Brazil. Anim. Conserv..

[37] Oficialdegui FJ, Sánchez MI, Monsalve-Carcaño C, Boyero L, Bosch J (2019). The invasive red swamp crayfish (*Procambarus clarkii*) increases infection of the amphibian chytrid fungus (*Batrachochytrium dendrobatidis*). Biol. Invas..

[38] Kupferberg SJ (2021). Seasonal drought and its effects on frog population dynamics and amphibian disease in intermittent streams. Ecohydrology.

[39] Kamoroff C (2020). Effective removal of the American bullfrog (*Lithobates catesbeianus*) on a landscape level: long term monitoring and removal efforts in Yosemite Valley Yosemite National Park. Biol. Invas..

[40] Vredenburg VT (2004). Reversing introduced species effects: Experimental removal of introduced fish leads to rapid recovery of a declining frog. Proc. Natl. Acad. Sci. U. S. A..

[41] Joseph MB, Knapp RA (2018). Disease and climate effects on individuals drive post-reintroduction population dynamics of an endangered amphibian. Ecosphere.

[42] Knapp RA, Briggs CJ, Smith TC, Maurer JR (2011). Nowhere to hide: Impact of a temperature-sensitive amphibian pathogen along an elevation gradient in the temperate zone. Ecosphere.

[43] Grasso RL (2023). Reproductive phenology of the California red-legged frog (*Rana draytonii*) in the Sierra Nevada of California, USA. Herpetol. Conserv. Biol..

[44] Adams AJ, Brown KC, Jennings MR, Grasso RL (2023). Homecoming or new pad: Historical evidence for California red-legged frogs and other amphibians in the Yosemite region California. Northwest. Nat..

[45] Stadtmann S, Seddon PJ (2018). Release site selection: Reintroductions and the habitat concept. Oryx.

[46] Bohmann K (2014). Environmental DNA for wildlife biology and biodiversity monitoring. Trends Ecol. Evol..

[47] Ficetola GF, Miaud C, Pompanon F, Taberlet P (2008). Species detection using environmental DNA from water samples. Biol. Lett..

[48] Rees HC, Maddison BC, Middleditch DJ, Patmore JRM, Gough KC (2014). REVIEW: The detection of aquatic animal species using environmental DNA – A review of eDNA as a survey tool in ecology. J. Appl. Ecol..

[49] Qu C, Stewart KA (2019). Evaluating monitoring options for conservation: Comparing traditional and environmental DNA tools for a critically endangered mammal. Sci. Nat..

[50] Bergman PS, Schumer G, Blankenship S, Campbell E (2016). Detection of adult green sturgeon using environmental DNA analysis. PloS one.

[51] Bedwell ME, Goldberg CS (2020). Spatial and temporal patterns of environmental DNA detection to inform sampling protocols in lentic and lotic systems. Ecol. Evol..

[52] Pope KL (2020). Designing environmental DNA surveys in complex aquatic systems: Backpack sampling for rare amphibians in Sierra Nevada meadows. Aquat. Conserv. Mar. Freshw. Ecosyst..

[53] Goldberg CS, Pilliod DS, Arkle RS, Waits LP (2011). Molecular detection of vertebrates in stream water: A demonstration using Rocky Mountain tailed frogs and Idaho giant salamanders. PloS ONE.

[54] Lopes CM (2020). Lost and found: Frogs in a biodiversity hotspot rediscovered with environmental DNA. Mol. Ecol..

[55] Carim KJ (2020). Environmental DNA sampling informs fish eradication efforts: Case studies and lessons learned. N. Am. J. Fish. Manag..

[56] Dejean T (2012). Improved detection of an alien invasive species through environmental DNA barcoding: The example of the American bullfrog Lithobates catesbeianus. J. Appl. Ecol..

[57] Ogata S, Doi H, Igawa T, Komaki S, Takahara T (2022). Environmental DNA methods for detecting two invasive alien species (American bullfrog and red swamp crayfish) in Japanese ponds. Ecol. Res..

[58] Hothem RL, Meckstroth AM, Wegner KE, Jennings MR, Crayon JJ (2009). Diets of three species of anurans from the Cache Creek Watershed, California, USA. J. Herpetol..

[59] Kupferberg SJ (1997). Bullfrog (*Rana catesbeiana*) invasion of a California river: The role of larval competition. Ecology.

[60] Balantic C (2021). Toward climate change refugia conservation at an ecoregion scale. Conserv. Sci. Pract..

[61] Lind AJ, Spinks PQ, Fellers GM, Bradley Shaffer H (2011). Rangewide phylogeography and landscape genetics of the Western U.S. endemic frog *Rana boylii* (Ranidae): Implications for the conservation of frogs and rivers. Conserv. Genet..

[62] California Department of Fish and Wildlife. California Natural Diversity Database (CNDDB) – Government version dated May 2020. (2020).

[63] California Department of Fish and Wildlife. Foothill Yellow-legged Frog Habitat Model for NSNF Connectivity - CDFW [ds1039]. (2014).

[64] Curtis AN, Tiemann JS, Douglass SA, Davis MA, Larson ER (2021). High stream flows dilute environmental DNA (eDNA) concentrations and reduce detectability. Divers. Distrib..

[65] Thomas AC, Howard J, Nguyen PL, Seimon TA, Goldberg CS (2018). eDNA Sampler: A fully integrated environmental DNA sampling system. Methods Ecol. Evol..

[66] Thomas AC, Nguyen PL, Howard J, Goldberg CS (2019). A self-preserving, partially biodegradable eDNA filter. Methods Ecol. Evol..

[67] Smith-Root. *Instruction manual: eDNA sampler. URL: *https://www.smith-root.com/support/downloads/edna-sampler-manual Accessed: 1 June 2020, <https://www.smith-root.com/support/downloads/edna-sampler-manual> (2019).

[68] Fellers GM, Kleeman PM, Miller DAW (2015). Wetland occupancy of pond-breeding amphibians in Yosemite National Park, USA. J. North Am. Herpetol..

[69] Halstead BJ, Kleeman PM, Rose JP, Fellers GM (2023). Sierra Nevada amphibians demonstrate stable occupancy despite precipitation volatility in the early 21st Century. Front. Ecol. Evol..

[70] Halstead BJ, Kleeman PM, Rose JP (2018). Time-to-detection occupancy modeling: An efficient method for analyzing the occurrence of amphibians and reptiles. J. Herpetol..

[71] Hyatt AD (2007). Diagnostic assays and sampling protocols for the detection of *Batrachochytrium dendrobatidis*. Dis. Aquat. Org..

[72] Wilcox TM (2013). Robust detection of rare species using environmental DNA: The importance of primer specificity. PloS ONE.

[73] Halstead BJ, Goldberg CS, Douglas RB, Kleeman PM, Ulrich DW (2020). Occurrence of a suite of stream-obligate amphibians in the timberlands of Mendocino County, California, examined using environmental DNA. Northwest. Nat..

[74] Strickler KM, Fremier AK, Goldberg CS (2015). Quantifying effects of UV-B, temperature, and pH on eDNA degradation in aquatic microcosms. Biol. Conserv..

[75] Boyle DG, Boyle DB, Olsen V, Morgan JAT, Hyatt AD (2004). Rapid quantitative detection of chytridiomycosis (*Batrachochytrium dendrobatidis*) in amphibian samples using real-time Taqman PCR assay. Dis. Aquat. Org..

[76] R Core Team. *R: A language and environment for statistical computing; version 4.2.1*, <https://www.R-project.org/> (2022).

[77] Microsoft Corporation. Microsoft Excel for Mac, version 16.81. (2024).

[78] (ESRI), E. S. R. I. ArcGIS Pro, version 2.8.6. (2022).

[79] Kamoroff C, Goldberg CS (2017). Using environmental DNA for early detection of amphibian chytrid fungus Batrachochytrium dendrobatidis prior to a ranid die-off. Dis. Aquat. Org..

[80] Pilliod DS, Goldberg CS, Arkle RS, Waits LP (2014). Factors influencing detection of eDNA from a stream-dwelling amphibian. Mol. Ecol. Resourc..

[81] Richards-Zawacki CL (2010). Thermoregulatory behaviour affects prevalence of chytrid fungal infection in a wild population of Panamanian golden frogs. Proc. R. Soc. B Biol. Sci..

[82] Puschendorf R (2011). Environmental refuge from disease-driven amphibian extinction. Conserv. Biol..

[83] Fellers, G. M. in *Amphibian Declines: The Conservation Status of United States Species* (ed M. J. Lannoo) 534–536 (University of California Press, 2005).

[84] U.S. Fish and Wildlife Service. Endangered and Threatened Wildlife and Plants; Threatened Species Status With Section 4(d) Rule for the Northwestern Pond Turtle and Southwestern Pond Turtle. 23534 (2024).

[85] Halstead BJ, Kleeman PM, Goldberg CS, Rose JP (2024). Comparison of two methods to detect the Northwestern Pond Turtle (*Actinemys marmorata*) and the invasive american bullfrog (*Lithobates catesbeianus*) in Interior Northern California. Chelonian Conserv. Biol..

[86] Moyle, P. B. Crawdads: Naturalized Californians. *California Water Blog* (2020).

[87] Hobbs, H. H. Crayfishes (Astacidae) of North and Middle America. *Biota of Freshwater Ecosystems Identification Manual 9, Environmental Protection Agency* (1972).

[88] Light T (2003). Success and failure in a lotic crayfish invasion: The roles of hydrologic variability and habitat alteration. Freshw. Biol..

[89] Elith J, Leathwick JR (2009). Species distribution models: Ecological explanation and prediction across space and time. Ann. Rev. Ecol. Evol. Syst..

[90] Curtis JA (2014). Incorporating cold-air pooling into downscaled climate models increases potential refugia for snow-dependent species within the Sierra Nevada ecoregion CA. PloS ONE.

[91] Millar CI (2015). Potential climatic refugia in semi-arid, temperate mountains: Plant and arthropod assemblages associated with rock glaciers, talus slopes, and their forefield wetlands, Sierra Nevada, California, USA. Quat. Int..

[92] Morelli TL (2020). Climate-change refugia: Biodiversity in the slow lane. Front. Ecol. Environ..

[93] Morelli TL (2016). Managing climate change refugia for climate adaptation. PloS ONE.

