# Supplementary material for: From eDNA to decisions using a multi-method approach to restoration planning in streams
Source: Sci Rep. 2024 Jun 21;14:14335. doi: 10.1038/s41598-024-64612-5 (PMC11192730; doi:10.1038/s41598-024-64612-5)
Supplement: Supplementary file 1 — Supplementary Information. [file 41598_2024_64612_MOESM1_ESM.pdf]

Supplementary Information

for:

From eDNA to decisions using a multi-method approach to restoration planning in streams

Adams, A.J.*[1, 2], C. Kamoroff [3, 4], N.R. Daniele [3], R.L. Grasso [3], B.J. Halstead [5], P.M. Kleeman [6], C. Mengelt [7], K. Powelson [8], T. Seaborn [9, 10], and C.S. Goldberg [1]

[1] School of the Environment, Washington State University, Pullman, Washington 99164 USA
[2] Earth Research Institute, University of California, Santa Barbara, California, 93106 USA
[3] Resource Management and Science, Yosemite National Park Service, California, 95318 USA
[4] Stillwater Sciences, Davis, California, 95618
[5] U.S. Geological Survey, Western Ecological Research Center, Dixon Field Station, Dixon, California 95620 USA
[6] U.S. Geological Survey, Western Ecological Research Center, Point Reyes Field Station, Point Reyes Station, California 94956 USA
[7] U.S. Geological Survey, Ecosystems Mission Area, Modoc Hall, Sacramento, California 95819 USA
[8] U.S. Forest Service, Tahoe National Forest, Nevada City, California 94949 USA
[9] School of Biological Sciences, Washington State University, Pullman, Washington 99164 USA
[10] School of Natural Resource Sciences, North Dakota State University, Fargo, North Dakota 58047 USA

*Corresponding author: Andrea J. Adams, andrea_adams@ucsb.edu

Any use of trade, product, or firm names is for descriptive purposes only and does not imply endorsement by the U.S. Government.

Table of contents

Supplementary Molecular Methods 3
Additional species surveys and assays 3
Northwestern pond turtle........ 3
Trout species........ 3
New *Pacifastacus leniusculus* species-specific assay 3
Supplementary Table S1. Validated assay for signal crayfish in the Yosemite National Park and Lake Tahoe regions. 4
eDNA assay quality control 5
Supplementary Decision Support Tool Information........ 6
Supplementary Box 1. 6
Menu of Conservation Actions 7
Supplementary Table S2. 9
Decision Support Tool Development........ 12
Literature Cited in Supplementary Information........ 13

Supplementary Molecular Methods

Additional species surveys and assays

In addition to *Rana boylii,* bullfrogs, and crayfish, one turtle species and three fish species were surveyed and assayed for as part of the present study.

Northwestern pond turtle

Turtles are among the most imperiled taxa in California [1,2]. The northwestern pond turtle (*Actinemys marmorata*), a species of special concern in the State of California, is proposed for U.S. Endangered Species Act listing [3] and has declined precipitously throughout its range. Like *R. boylii*, *A. marmorata* can be an indicator of ecosystem health, and shares many of the same threats as *R. boylii*, including habitat alteration/conversion, pathogens, drought, and non-native species [1,4,5].

The assay for *A. marmorata* [6] followed the protocols in the main text. We did not assay for *A. marmorata* in the 2021 samples due to funding constraints; therefore, they were not included in the site prioritization analysis. We detected *A. marmorata* at four more streams with environmental DNA (eDNA) than with visual encounter surveys (VESs) (eDNA = 9; VES = 5). This indicates that, in our study area, eDNA is a more sensitive method for *A. marmorata* detection than VES.

Trout species

Although *R. boylii* have coevolved and are sympatric with native fish throughout their range, some Sierra Nevada amphibian species are susceptible to trout predation when the fish are introduced from outside of their native range [7,8]. Three trout species occur within the range of *R. boylii* in the southern Sierra Nevada: brown trout (*Salmo trutta*; introduced); rainbow trout (*Oncorhynchus mykiss*; native and introduced) and brook trout (*Salvelinus fontinalis*; introduced).

Assays for these species followed the protocols described in the main text: brown trout (*S. trutta* [9]), rainbow trout (*O. mykiss* [10]), and brook trout (*S. fontinalis* [11]). In 2020, both *O. mykiss* and *S. trutta* were widely detected across our sites with eDNA, and these species frequently co-occurred; therefore, we did not assay for these species in the 2021 samples when faced with funding constraints.

New *Pacifastacus leniusculus* species-specific assay

Because of the high sequence diversity for *P. leniusculus*, we sequenced samples from the Sierra Nevada to design a regional assay. We extracted DNA from 79 samples collected in the Lake Tahoe region using soft tissue from within the cephalothorax or leg for sequencing and using a DNeasy Blood & Tissue Kit (Qiagen, Inc., Hilden, Germany). We then amplified fragments of the COI mitochondrial gene using the primers

LCOI1490 and HCO2198 [12]. Each PCR reaction included 3 µL of DNA extract in a total volume of 15 µL. Reactions consisted of 1X Multiplex PCR Mix (Qiagen Inc.) and 0.4 µL of each primer on a Bio-Rad C1000 Touch thermocycler. Reactions were activated for 15 minutes at 95°C then run for 50 cycles of 94°C for 30 seconds, followed by 50°C for 90 seconds, followed by 72°C for 60 seconds, and with a final extension of 60°C for 30 minutes. We checked all samples on a 2% agarose gel to confirm amplification. Samples were cleaned using ExcelaPure PCR purification blocks (Edge BioSystems, San Jose, California, USA) and Sanger sequenced at the Arizona Genetics Core (Tucson, Arizona, USA) on a 3730 DNA Analyzer (Applied Biosystems).

From these sequences, we created a consensus sequence using Sequencher v5.3 (Gene Codes Corp., Ann Arbor, Michigan, USA). Due to high levels of genetic variation, we considered genetic regions for qPCR primers for the eDNA assay where similarity was greater than 90%. We then used Primer Express 3.0.1 (Applied Biosystems, Foster City, California, USA) to create assay designs and validated them *in silico* using Primer-BLAST [13]. For the primer/probe set that passed this validation (Table S1), we further validated it using DNA extracted from tissue samples. For this validation, each qPCR plate included 3 µL of DNA extract in a total volume of 15 µL. Reactions consisted of 1X QuantiTect Multiplex PCR Mix (Qiagen Inc.), with 0.2 µM of each primer and probe on a Bio-Rad CFX96 Touch Real-Time PCR Detection System. To test for inhibition of the qPCR, each well included an exogenous internal positive control (IC; Qiagen product 211454). Reactions activated for 15 minutes at 95°C then ran for 50 cycles of 94°C for 60 seconds followed by 60°C for 60 seconds. We validated the assay using 10 tissue samples from the Lake Tahoe region and 20 from the Merced River in the Yosemite region. The assay amplified for all samples. We tested assay specificity against co-occurring invasive Astacidae: red swamp crayfish (*Procambarus clarkii*; 6 individuals), and the virile crayfish (*Orconectes virilis*; 5 individuals); none of the non-target crayfish species amplified. We further developed a standard curve down to a 1:1,000,000 dilution of tissue sample, which amplified in all reactions, and tested eDNA samples collected from a known field site with signal crayfish, which tested positive.

Supplementary Table S1. Validated assay for signal crayfish in the Yosemite National Park and Lake Tahoe regions.

Forward Primer	GCGGCTATTGCTCATGCA
Reverse Primer (5’-3’)	AACACCCGCTAAATGAAGTGAAA
Probe	FAM-CTTCTGTTGATTTAGGAATT-MGBNFQ

Signal crayfish assay acknowledgements. The signal crayfish assay was developed under funding provided by the USDA Forest Service, Pacific Southwest Research Station on a project led by Karen Pope and Jonah Piovia-Scott. John Umek provided samples from the Lake Tahoe region. Field samples for validation were collected by Nicolette Nelson.

eDNA assay quality control

We evaluated the limit of detection (LOD) for all assays applied in this study using gblocks (Integrated DNA Technologies, Inc., Coralville, Iowa, USA), defined as the lowest copy number where >95% of reactions amplify. This was between 5 and 10 copies for all assays except for the American bullfrog (*Lithobates catesbeianus*; hereafter "bullfrogs"), where it was <5 copies. Reaction efficiencies were 85-110% except for rainbow trout, where efficiencies were between 61.5 and 85.3%. The r^2 values of all standard curves were >0.98. Standard curves for American bullfrogs, western pond turtles, brown trout, and signal crayfish were constructed using diluted DNA extracted from tissue samples; standard curves for the remaining species were made using gblocks.

Supplementary Decision Support Tool Information

Supplementary Box 1.
Restoration status categories for current or potential *Rana boylii* sites.

A - *R. boylii* present without non-native predators;

B - *R. boylii* present with non-native predators (bullfrogs or crayfish);

C - *R. boylii* absent without non-native predators;

D - *R. boylii* absent with non-native predators (bullfrogs or crayfish).

Menu of Conservation Actions

Actions are categorized into six interdependent actions. This menu of actions is linked to specific status categories from Supplementary Box 1 and the decision tree in Fig. 3 in the main manuscript.

1 – HABITAT ASSESSMENTS (Appropriate for status categories C, D). Historically occupied habitat does not necessarily constitute currently suitable habitat. Conduct *R. boylii* habitat suitability assessments in places where *R. boylii* are absent, but the site has been identified as potentially suitable habitat because of historical *R. boylii* occurrence. This is a prerequisite to all other actions if habitat suitability is unknown. The Sierra Nevada encompasses an inherently dynamic landscape; therefore, include climate change considerations in habitat assessments for long-term planning.

2 – DEMOGRAPHICS/BASELINE MONITORING (Appropriate for status categories A, B). Knowledge of demography guides reasonable expectations for reintroduction outcomes and aids in the design of more successful reintroduction programs. Where *R. boylii* are present, egg mass counts and other measures of demographic rates (e.g. capture-mark-recapture) can be estimated to provide a baseline for that population and determine its status as a potential source for translocations. Egg mass surveys for *R. boylii* can quantify changes in abundance and survival and estimate other vital rates in both (a) at-risk populations and (b) larger, presumably stable populations over several years. The results of (b) would help to identify potential donor sites for egg mass translocations to at-risk or extirpated sites. Then changes in vital rates can be compared between the two, to determine if stable populations are affected by egg mass translocation away from these sites, and whether *in situ* rearing assists at-risk populations.

3 – BULLFROG & CRAYFISH CONTROL (Appropriate for status categories B, D). Conduct bullfrog control during spring and summer at sites with suitable *R. boylii* habitat where bullfrogs were detected, and where sufficient barriers are present to inhibit reinvasion. Conduct crayfish control in sites with suitable *R. boylii* habitat where crayfish were detected, and where feasible based on the presence of dispersal barriers. Prioritize bullfrog and crayfish control first at places where *R. boylii* occur or where these species are in proximity to *R. boylii* populations.

4 – SPECIES DETECTIONS. (Appropriate for new sites with unknown status, and sites where species status is unclear due to limited sampling). This action is for areas where more information is necessary to determine which species are present. Visual encounter surveys and eDNA sampling can facilitate the continued monitoring of *R. boylii*-occupied sites for the invasion of non-native species like bullfrogs and crayfish. Target the timing of eDNA surveys when

tadpoles are most likely to be in the system (mid-May to early August). In an average year, peak flows are usually around May 1, and most creek flows are decreasing at this time because little flow remains in the tributaries. The main-stem rivers are different with higher elevation snowpack and late-season storms, so these can be surveyed later in the season.

5 – CAPTIVE PROPAGATION (EITHER *IN SITU* REARING OR CAPTIVE PROPAGATION FOLLOWED BY RELEASE). (Appropriate for status category A). In places where egg mass or other vital rate data are available to serve as a baseline, *in situ* rearing or captive propagation at a qualified facility, followed by release to the collection site, may be conducted. Conduct *in situ* rearing and releases when suitable habitat free of non-native predators has been established. All necessary permits and permissions would need to be obtained from regulatory agencies prior to conducting *in situ* rearing, captive propagation, and release.

6 – REINTRODUCTIONS/TRANSLOCATIONS (Appropriate for status category A, or C if suitable habitat). Once *R. boylii* demographics at potential source sites have been established, and non-native predator eradication has been achieved, reintroductions and/or translocations may be considered. Adherence to strict guidelines will ensure pathogens are not translocated in this process, and, like *in situ* rearing, translocations and reintroductions will require approvals from appropriate regulatory agencies.

Supplementary Table S2.

Potential management actions by site. Descriptions of management actions are provided in this Supplementary Information above and in the text. HA = habitat assessments; DG = demographics/baseline monitoring; BC = bullfrog or crayfish control; CP = Captive propagation (either *in situ* or *ex situ* rearing followed by release); SD = species detection monitoring (i.e., continued environmental DNA [eDNA] and visual encounter surveys [VESs]); TR = translocations. High priority sites are indicated in red, moderate priority in yellow, and low priority in green. Priority designations are informed by their status (refer to Supplementary Box 1 and Column 2 of this table); however, the circumstances unique to each site determine level of priority (refer to the main text for a description of priority designations and examples).

Site	Status	Mgmt. action(s)	Comments
**Merced River Watershed (North Fork)**			
**Bean Creek**	D	BC	Bullfrogs and crayfish detected. No barriers to dispersal to nearby *R. boylii* sites Merced 01 and Merced 02. High priority to control bullfrogs and crayfish nearest the *R. boylii*-occupied sites.
**Deer Lick Creek**	C	HA	No bullfrogs or crayfish detected. Evaluate if suitable for reintroduction with habitat assessments.
**Jordan Creek**	D	BC	Bullfrogs detected. No barriers to dispersal to two nearby *R. boylii*-occupied sites. High priority to control bullfrogs and crayfish nearest the *R. boylii*-occupied sites.
**Merced 01, Merced 02**	A	DG; SD	Demographic data needed on *R. boylii* population to determine if captive propagation is warranted. Population likely threatened by nearby bullfrogs and crayfish in two nearby creeks. Continue sampling (species detections) for bullfrogs and crayfish in order to monitor for invasions into this site.
**Merced River Watershed (Main Stem)**			
**Avalanche Ck., Bridalveil Ck., Bull Ck., Cold Canyon Ck., Indian Ck., Moss Ck., Snow Ck., Sweetwater Ck.**	C	HA	No bullfrogs or crayfish detected. Evaluate which of these would be suitable sites for reintroduction with habitat assessments.
**Bear Creek**	D	HA	Crayfish detected near confluence with Merced. Waterfall may be a barrier to dispersal to upper reaches, where no species were detected. Evaluate upper reaches for *R. boylii* habitat suitability.
**Cascade Creek**	D	HA	Crayfish detected. May be assessed for crayfish control, but this site is not in proximity to other *R. boylii* sites.

Site	Status	Mgmt. action(s)	Comments
**El Portal**	D	BC, HA	Only crayfish detected; no bullfrogs. Gorge at the National Park Service boundary provides a likely dispersal barrier; no other barriers occur on the main stem of the Merced River. Evaluate for feasibility of crayfish control.
**Fern Spring**	C	None	No species detected. Site of historical *R. boylii* observation in Yosemite Valley; not currently a suitable reintroduction site given that it is a high-traffic area.
**Halls Gulch**	D	HA; BC	Bullfrogs and crayfish detected. This site is likely the largest bullfrog breeding area on the Merced River between Yosemite National Park and Lake McClure. No onsite bullfrog dispersal barriers; however, there are crayfish barriers for the first 800 m above the confluence. Possibly more barriers upstream.
**Merced 03**	C	TR	No species detected. Suitable *R. boylii* habitat present. Suitable translocation recipient site.
**Tenaya Creek, Yosemite Creek**	D	BC	Crayfish detected. Evaluate whether crayfish control would be feasible here.
**Merced River Watershed (South Fork)**			
**Alder Ck., Bishop Ck., Iron Ck., Mosquito Ck., Owl Ck., Rush Ck., Squirrel Ck., Strawberry Ck.**	C	HA	No bullfrogs or crayfish detected. Habitat assessments may determine whether sites would be suitable for reintroduction.
**Big Ck., Laurel Ck., Skelton Ck., South Fork Merced River**	D	HA	Bullfrogs and crayfish detected. Habitat assessments could be conducted to assess feasibility of control.
**Tuolumne River Watershed (Main Stem)**			
**Big Jackass Creek**	D	BC, HA	Crayfish detected. In proximity to *R. boylii*-occupied site Tuolumne 06; evaluate feasibility of crayfish control and implement if possible.
**Cherry Ck., Corral Ck., Eleanor Ck., Frog Ck., Indian Ck., Jawbone Ck.**	C	HA	No bullfrogs or crayfish detected. Evaluate whether any sites would be suitable for *R. boylii* translocation with habitat assessments.
**Tuolumne 01, Tuolumne 02, Tuolumne 05**	A	DG	Demographic data needed on *R. boylii* populations at these sites to determine suitability as source populations or whether captive propagation or *in situ* rearing are needed to bolster existing populations first.
**Tuolumne 03**	A	DG	Demographic data needed on *R. boylii* population. Not an ideal site to serve as a source population because of its remote location.

Site	Status	Mgmt. action(s)	Comments
**Tuolumne 04**	B	BC; DG; CP, SD	Small breeding *R. boylii* population present[1]. Bullfrogs detected in 2021. Natural fish barrier occurs ~1 km upstream. Prioritize bullfrog control and continue species monitoring to evaluate progress of control.
**Tuolumne 06**	B	HA; BC; DG	Crayfish detected and *R. boylii* present. Evaluate site for feasibility of crayfish control with habitat assessments, and prioritize *R. boylii* demographic monitoring. Monitoring could provide insight into dynamics of co-occurrence between *R. boylii* and crayfish.
**Tuolumne River Watershed (Middle)**			
**Evergreen Road, Plum Creek**	C	HA	No bullfrogs or crayfish detected. Evaluate whether this would be a suitable site for reintroduction with habitat assessments.
**Tuolumne 07**	B	HA; DG; BC	Crayfish but no bullfrogs detected. Habitat assessment may determine whether crayfish control is feasible. High priority with crayfish and *R. boylii* in proximity/co-occurring. Collect *R. boylii* demographic data.
**Tuolumne 08**	B	BC; SD; DG	Bullfrogs detected for the first time in 2021 at this *R. boylii*-occupied site. Prioritize bullfrog control and monitor with more species detections, conduct *R. boylii* demographic assessments.
**Tuolumne River Watershed (North Fork)**			
**Basin Ck.**	C	HA	No species detected. Evaluate whether this would be a suitable site for reintroduction with habitat assessments.
**Tuolumne 10**	B	BC; HA; DG	Both *R. boylii* and crayfish detected. Evaluate site for feasibility of crayfish control. Conduct demographic monitoring on *R. boylii* population.
**Tuolumne River Watershed (South Fork)**			
**Ackerson Ck., Carlon Falls, Hardin Flat, Soldier Ck.**	C	HA	No species detected. Evaluate whether this would be a suitable site for reintroduction with habitat assessments.
**Tuolumne 09**	B	HA; BC; DG	*R. boylii* and crayfish present. Evaluate whether crayfish control is feasible. Collect *R. boylii* demographic data. Monitoring could provide insight into dynamics of co-occurrence between *R. boylii* and crayfish.

[1] *Rana boylii* tadpoles have recently been observed at Tuolumne 04, but not as part of the present study. Therefore, for the purposes of management action planning and prioritization, this site is considered occupied. Because there were no *R. boylii* detections as part of this study, *R. boylii* are not indicated in Fig. 2 of the main text.

Decision Support Tool Development

A systematic approach to prioritizing restoration sites and selecting potential management actions reduces uncertainty in the decision-making process.

Habitat assessments are necessary when a site is unoccupied by *R. boylii* to determine if it is suitable for reintroductions. Habitat assessments may also be appropriate when non-native predators are present to determine whether eradication is attainable to avoid investing in eradication projects that will not be successful; for example, if reinvasion risk is high from outlying areas post-removal. Habitat structure and complexity can play a large role in the success of an eradication program, as was the case with the eradication of bullfrogs from Yosemite Valley [15].

Demographics (including age-stage structure, sex, and counts [16]) and baseline monitoring are needed when *R. boylii* is present, to determine (a) whether *in situ* or captive rearing could be used to bolster the existing population, or (b) whether the population is robust enough to serve as a source for translocations elsewhere. Bullfrog and crayfish eradication are considered a high priority at sites where *R. boylii* are already present.

Bullfrog and crayfish control may also be considered at sites with suitable habitat or that are within dispersal distance of existing *R. boylii*-occupied sites, but these are a lower priority than where *R. boylii* and these non-native aquatic predators co-occur. Species detections are important at sites that have *R. boylii* but are within dispersal distance of bullfrogs and crayfish, in order to monitor for new invasions (a task for which eDNA sampling is highly suited; refer to main text).

At sites where demographic and baseline monitoring are already underway, captive propagation may be considered. Captive propagation includes *in situ* rearing (collecting animals and rearing them in pens in the stream at or near the collection site) as well as *ex situ* rearing (captive rearing at a qualified facility such as a zoo). Both *in situ* and *ex situ* rearing are followed by release to the site of collection, as compared to reintroductions and translocations.

Reintroductions and/or translocations may be considered at sites that are already known to be suitable habitat and are free of non-native aquatic predators. In many cases, multiple conservation actions may be considered at any particular site (Supplementary Table S2).

Literature Cited in Supplementary Information

1 Jennings, M. & Hayes, M. P. Amphibian and reptile species of special concern in California. 255 (California Department of Fish and Game, Rancho Cordova, California, 1994).

2 Thomson, R. C. *California amphibian and reptile species of special concern*. (University of California Press, 2016).

3 U.S. Fish and Wildlife Service. Endangered and Threatened Wildlife and Plants; Threatened Species Status With Section 4(d) Rule for the Northwestern Pond Turtle and Southwestern Pond Turtle. 23534 (2024).

4 Bury, R. B. & Germano, D. J. in *Conservation Biology of Freshwater Turtles and Tortoises: A Compilation Project of the IUCN/SSC Tortoise and Freshwater Turtle Specialist Group. Chelonian Research Monographs* Vol. 5 (eds A.G.J. Rhodin *et al.*) 001.001-001.009 (2008).

5 Manzo, S. *et al.* Conservation of northwestern and southwestern pond turtles: Threats, population size estimates, and population viability analysis. *Journal of Fish and Wildlife Management* **12**, 485-501, doi:10.3996/jfwm-20-094 (2021).

6 Kamoroff, C. *et al.* Investigating aquatic species distributions for Sequoia and Kings Canyon National Parks: A comparison of visual and environmental DNA surveys in streams. *Natural Areas Journal* **43**, 225-234, doi:10.3375/0885-8608-43.4.225 (2023).

7 Knapp, R. A. & Matthews, K. R. Non-native fish introductions and the decline of the mountain yellow-legged frog from within protected areas. *Conservation Biology* **14**, 428-438 (2000).

8 Wilkins, L. G. E., Matthews, K. R., Steel, Z. L., Nusslé, S. C. & Carlson, S. M. Population dynamics of *Rana sierrae* at Dusy Basin: Influence of non-native predators, drought, and restoration potential. *Ecosphere* **10**, e02951, doi:10.1002/ecs2.2951 (2019).

9 Carim, K. J. *et al.* An environmental DNA marker for detecting nonnative brown trout (*Salmo trutta*). *Conservation Genetics Resources* **8**, 259-261, doi:10.1007/s12686-016-0548-5 (2016).

10 Brandl, S. *et al.* Ten real-time PCR assays for detection of fish predation at the community level in the San Francisco Estuary–Delta. *Molecular Ecology Resources* **15**, 278-284, doi:https://doi.org/10.1111/1755-0998.12305 (2015).

11 Wilcox, T. M. *et al.* Robust detection of rare species using environmental DNA: The importance of primer specificity. *PloS one* **8**, e59520, doi:10.1371/journal.pone.0059520 (2013).

12 Folmer, O. DNA primers for amplification of mitochondrial cytochrome c oxidase subunit I from diverse metazoan invertebrates. *Mol Mar Biol Biotechnol*, 294-299 (1994).

13 Ye, J. *et al.* Primer-BLAST: A tool to design target-specific primers for polymerase chain reaction. *BMC Bioinformatics* **13**, 134, doi:10.1186/1471-2105-13-134 (2012).

14 Brown, C. *et al.* Using demography to evaluate reintroductions for conservation of the endangered frog, *Rana sierrae*, in streams. *Herpetologica* **76**, 383-395 (2020).

15 Kamoroff, C. *et al.* Effective removal of the American bullfrog (*Lithobates catesbeianus*) on a landscape level: long term monitoring and removal efforts in Yosemite Valley, Yosemite National Park. *Biological Invasions* **22**, 617-626, doi:10.1007/s10530-019-02116-4 (2020).
16 Skalski, J. R., Ryding, K. E. & Millspaugh, J. *Wildlife demography: Analysis of sex, age, and count data*. (Elsevier, 2010).
